# Mindfulness, Attention, and Impulsivity in Bipolar Disorder

**DOI:** 10.1192/j.eurpsy.2023.262

**Published:** 2023-07-19

**Authors:** N. E. Fares-Otero, B. Solé, S. Martin-Parra, F. Piazza, J. Sanchez-Moreno, E. Vieta, A. Martinez-Aran

**Affiliations:** Psychiatry and Psychology, Bipolar and Depressive Disorders Unit, Hospital Clínic of Barcelona, Institute of Neurosciences, Barcelona, Spain

## Abstract

**Introduction:**

Bipolar disorder (BD) is a chronic mental disorder characterized by mood instability^1^. BD is further related to neurocognitive and functional disruptions that remain remarkably stable even when patients are euthymic, leading to poor well-being and quality of life. Mindfulness means paying attention on purpose, in the present moment, and involves different facets such as observing, describing, acting with awareness, non-judging and non-reacting of inner experience. It remains unclear whether mindfulness and its specific facets are differentially associated with different aspects of attention and trait impulsivity in individuals with BD.

**Objectives:**

To examine associations between different mindfulness facets, and different aspects of attention and trait impulsivity in BD.

**Methods:**

This study was approved by the Hospital Clínic Ethics and Research Board (HCB/2017/0432). After informed consent, 94 outpatients, *M* age = 45.57, *SD =* 9.8, range 19-61 years, 41.5% Male, 63.8% BD-I according to DSM-5 criteria, in partial or total remission based on Young Mania Rating Scale (YMRS; *M* = 1.81, *SD* = 2.11) and Hamilton Depression Rating Scale (HDRS; *M* = 5.46, *SD* = 3.71) were enrolled in this study. Participants were evaluated using the Five Facet Mindfulness Questionnaire (FFMQ) to assess Mindfulness, the Trail Making Test (TMT-A) and the Conner’s Continuous Performance test (CPT-II) to assess Attention, and the Barratt Impulsiveness Scale (BIS-11) to assess Impulsivity. Pearson correlations were performed, and statistical significance was evaluated two-sided at the 5% threshold.

**Results:**

Mindfulness-Describing was negatively associated with Cognitive and Non-Planning Impulsivity (*r* = -.43 and -.28, *p* < .001), Mindfulness-Acting with Awareness was negatively associated with Cognitive, Motor and Non-Planning Impulsivity (*r* = -.27 to -.45, *p* < .001), Mindfulness Non-Judging (*r* = -.33 and -.34, *p* < .001) and Non-Reacting (*r* = -.30 and -.46, *p* < .001) of inner experience were negatively associated with Cognitive and Motor Impulsivity. No associations were found between neither Mindfulness nor Impulsivity with any aspects of Attention.

**Conclusions:**

Preliminary findings suggest that better performance in specific facets of mindfulness (describing, acting with awareness, non-judging or reacting of inner experience) may be related to a decrease in different aspects of trait impulsivity. Further longitudinal and interventional research is needed on underlying mechanisms. Nonetheless, our study suggests the need for including mindfulness-based approaches to improve behavioral and functional outcomes for those with BD.

**Funding:**

This work was supported by the European Union Horizon 2020 research and innovation program (EU.3.1.3. Treating and managing disease: Grant 945151), CIBERSAM, FIS PI17/00941 ISCIII, European Regional Development Fund.

**References:**

1. Carvalho AF, Firth J, Vieta E. Bipolar Disorder. *N Engl J Med.* 2020;383(1):58-66. doi:10.1056/NEJMra1906193

**Disclosure of Interest:**

None Declared

